# Supplementary material for: Altered microRNA Transcriptome in Cultured Human Liver Cells upon Infection with Ebola Virus
Source: Int J Mol Sci. 2021 Apr 6;22(7):3792. doi: 10.3390/ijms22073792 (PMC8038836; doi:10.3390/ijms22073792)
Supplement: Supplementary file 1 [file ijms-22-03792-s001.zip › Supplementary_File/C_ GO_Analysis_Results/16-30nt_go_Reston-96h-Huh7_vs_Control-96h-Huh7_up.mature_mirna_targets/CC_Pvalue_tree.pdf]

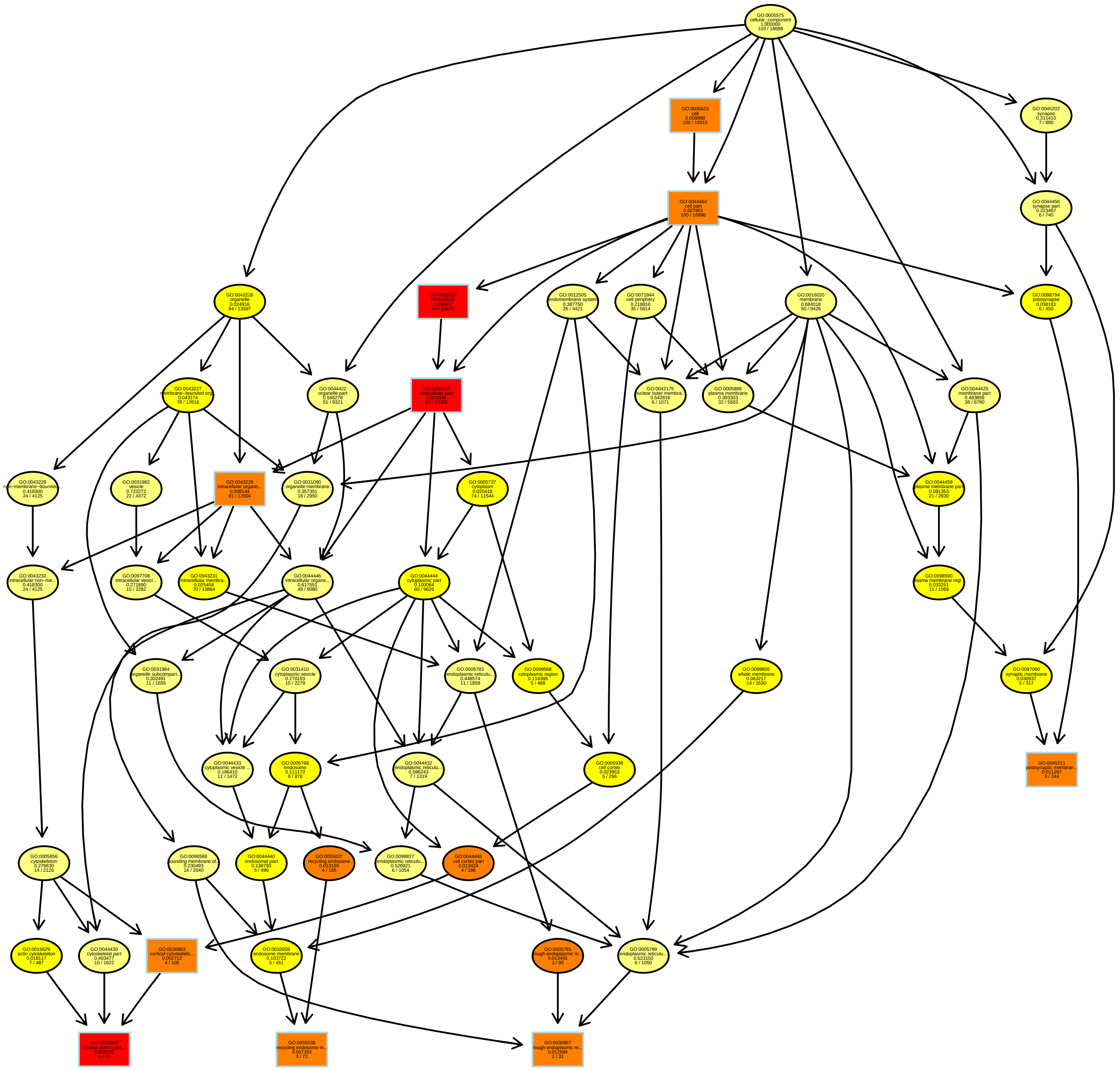
GO:0005575 cellular_component 1.000000 103 / 18698
GO:0005623 cell 0.008988 100 / 16915
GO:0045202 synapse 0.211410 7 / 880
GO:0044464 cell part 0.007901 100 / 16886
GO:0044456 synapse part 0.223467 6 / 740
GO:0043226 organelle 0.024916 84 / 13597
GO:0005622 intracellular 0.000457 94 / 14673
GO:0012505 endomembrane system 0.387750 26 / 4421
GO:0071944 cell periphery 0.218816 35 / 5614
GO:0016020 membrane 0.684018 50 / 9426
GO:0098794 postsynapse 0.038181 6 / 450
GO:0043227 membrane−bounded org... 0.043174 78 / 12616
GO:0044422 organelle part 0.566278 51 / 9321
GO:0044424 intracellular part 0.001058 92 / 14395
GO:0042175 nuclear outer membra... 0.542816 6 / 1071
GO:0005886 plasma membrane 0.393303 32 / 5503
GO:0044425 membrane part 0.483856 38 / 6780
GO:0043228 non−membrane−bounded... 0.418300 24 / 4125
GO:0031982 vesicle 0.722272 22 / 4372
GO:0043229 intracellular organe... 0.008144 81 / 12604
GO:0031090 organelle membrane 0.357351 18 / 2950
GO:0005737 cytoplasm 0.020418 74 / 11544
GO:0044459 plasma membrane part 0.091353 21 / 2830
GO:0043232 intracellular non−me... 0.418300 24 / 4125
GO:0097708 intracellular vesicl... 0.271890 15 / 2282
GO:0043231 intracellular membra... 0.025404 70 / 10864
GO:0044446 intracellular organe... 0.617551 49 / 9080
GO:0044444 cytoplasmic part 0.100064 60 / 9626
GO:0098590 plasma membrane regi... 0.033251 11 / 1069
GO:0031984 organelle subcompart... 0.302491 11 / 1655
GO:0031410 cytoplasmic vesicle 0.270193 15 / 2279
GO:0005783 endoplasmic reticulu... 0.448574 11 / 1859
GO:0099568 cytoplasmic region 0.116395 5 / 468
GO:0098805 whole membrane 0.063217 14 / 1630
GO:0097060 synaptic membrane 0.030937 5 / 317
GO:0044433 cytoplasmic vesicle ... 0.186410 11 / 1472
GO:0005768 endosome 0.111172 8 / 878
GO:0044432 endoplasmic reticulu... 0.596243 7 / 1319
GO:0005938 cell cortex 0.023953 5 / 296
GO:0045211 postsynaptic membran... 0.011297 5 / 244
GO:0005856 cytoskeleton 0.279630 14 / 2126
GO:0098588 bounding membrane of... 0.230493 14 / 2040
GO:0044440 endosomal part 0.138793 5 / 496
GO:0055037 recycling endosome 0.013155 4 / 165
GO:0098827 endoplasmic reticulu... 0.526921 6 / 1054
GO:0044448 cell cortex part 0.013424 4 / 166
GO:0015629 actin cytoskeleton 0.018117 7 / 487
GO:0044430 cytoskeletal part 0.403477 10 / 1622
GO:0030863 cortical cytoskeleto... 0.002712 4 / 105
GO:0010008 endosome membrane 0.103722 5 / 451
GO:0005791 rough endoplasmic re... 0.013491 3 / 90
GO:0005789 endoplasmic reticulu... 0.523150 6 / 1050
GO:0030864 cortical actin cytos... 0.000782 4 / 75
GO:0055038 recycling endosome m... 0.007353 3 / 72
GO:0030867 rough endoplasmic re... 0.012594 2 / 31
